# Thermal Oxidative Aging and Service Life Prediction of Commercial Ethylene–Propylene–Diene Monomer Spacer Damping Composites for High–Voltage Transmission Lines

**DOI:** 10.3390/polym16091186

**Published:** 2024-04-24

**Authors:** Yutong Zhou, Lvchao Qiu, Zongchao Xu, Shixuan Huang, Jingkai Nie, Hang Yin, Feng Tu, Zhoufeng Zhao

**Affiliations:** 1 State Grid Zhejiang Electric Power Co., Ltd. Research Institute, Hangzhou 310014, China; 2 State Grid Smart Grid Research Institute Co., Ltd., Beijing 102209, China; niejingkai@geiri.sgcc.com.cn (J.N.); yinhang@geiri.sgcc.com.cn (H.Y.); 3 State Grid Zhejiang Electric Power Co., Ltd., Hangzhou 310007, China

**Keywords:** spacer bars, damping property, rubber composites, thermal oxidative aging, life prediction

## Abstract

The aging behavior and life prediction of rubber composites are crucial for ensuring high-voltage transmission line safety. In this study, commercially available ethylene–propylene–diene monomer (EPDM) spacer composites were chosen and investigated to elucidate the structure and performance changes under various aging conditions. The results showed an increased C=O peak intensity with increasing aging time, suggesting intensified oxidation of ethylene and propylene units. Furthermore, the surface morphology of commercial EPDM composites displayed increased roughness and aggregation after aging. Furthermore, hardness, modulus at 100% elongation, and tensile strength of commercial EPDM composites exhibited a general increase, while elongation at break decreased. Additionally, the damping performance decreased significantly after aging, with a 20.6% reduction in loss factor (20 °C) after aging at 100 °C for 672 h. With increasing aging time and temperature, the compression set gradually rose due to the irreversible movement of the rubber chains under stress. A life prediction model was developed based on a compression set to estimate the lifetime of rubber composites for spacer bars. The results showed that the product’s life was 8.4 years at 20 °C. Therefore, the establishment of a life prediction model for rubber composites can provide valuable technical support for spacer product services.

## 1. Introduction

Rubber composites play a critical role in the fields of sealing and protection, vibration and noise reduction, insulation of power transmission, and transformation equipment, owing to their excellent barrier, viscoelastic, and mechanical properties [1,2]. The spacer bar is a crucial component installed on high-voltage transmission lines, comprising metal clamps and rubber damping materials. Based on the damping and energy dissipation characteristics of rubber composites, spacer devices can suppress the whipping of wire bundles caused by strong winds and icing [3]. However, the performance of rubber materials gradually degrades over time due to exposure to light, oxygen, and heat, consequently diminishing the service life of products. Therefore, research on the aging behavior of rubber materials has always been a critical issue to the reliable service of rubber materials.

The aging of rubber composites is extremely complex, and most research studies on rubber aging behavior primarily focus on understanding the underlying aging mechanism [4]. Exploring the aging mechanism of rubber materials serves as a guiding principle for optimizing and enhancing rubber material formulations, while the study of rubber aging life aids in predicting the service life of rubber products. This proactive approach enables the timely replacement of products nearing the end of service life, thereby averting potential safety hazards arising from product failure. There are many factors affecting the aging of rubber materials, including rubber matrix, types, and amounts of anti-aging agents, fillers, and vulcanization systems [5,6]. Among these factors, the type of rubber matrix is the most important factor affecting the aging behavior of rubber. Unsaturated rubber molecular chains exhibit limited stability, rendering them susceptible to thermal oxygen reactions, which can result in chain scission. In comparison to other commonly used rubbers such as natural rubber, butadiene–styrene rubber, and butadiene rubber, ethylene–propylene–diene copolymer rubber (EPDM) demonstrates a higher molecular chain saturation and remarkable antioxidant capabilities. As a result, EPDM has emerged as the preferred material for outdoor spacer rubber applications. However, the third monomer utilized for vulcanization crosslinking in EPDM exhibits sensitivity towards thermal oxygen, potentially leading to molecular chain breakage and aging.

The artificially accelerated aging test serves as a crucial technique for examining the durability of rubber composites. Since the 1940s, stress relaxation experiments have been a component of studying the aging process of rubber composites [7]. Tobolsky and his coworkers were the first to use stress relaxation to study the aging behavior of rubber materials [8]. Subsequently, researchers studied the stress relaxation of specimens under uniaxial tensile, compressive, or shear loads [9,10]. The uniaxial tensile loading mode has received great attention in the literature and is considered the most suitable method for basic research. However, compression stress relaxation experiments are the most precise and appropriate approach for assessing the properties of rubber composites utilized in sealing and shock absorption applications [11]. The aging performance changes of rubber materials are related to their physical and chemical structure interactions. The contribution of physical interactions mainly includes conformational changes caused by the movement of rubber molecular chains, changes in filler network structure. The chemical reaction is the breaking of chemical crosslinking bonds caused by the thermal oxidation of rubber molecular chains. The chemical bonds breaking form new active molecular chain ends, and stress is released. The molecular chains will form new crosslinking structures, leading to irreversible stress relaxation and permanent deformation of the sample. Under high temperatures and long-term aging conditions, changes in chemical structure dominate over changes in physical structure [12]. Previous studies have investigated the aging mechanism and anti-aging methods of EPDM composites. Zhao et al. found that with the increase in aging time, the content of hydroxyl and carbonyl groups as well as the carbonyl index increased, and vulcanized EPDM gradually oxidized and degraded. Consequently, the vulcanized EPDM chains underwent a limited conformational transformation. These irreversible changes, such as molecular chain breakage and crosslinking, affected the physical properties of EPDM materials, ultimately altering their hardness, strength, and loss factor [13]. Further investigations by Delor-Jestin et al. demonstrated that during thermal oxidative aging of EPDM composites, the elongation at break decreased with increasing aging time, and the tensile strength decreased by 25% after 1000 h of aging. In terms of dynamic performance, the peak of tan δ decreased with increasing aging time, with the corresponding temperature shifted towards a higher temperature region [14]. CHOI et al. conducted a study on the hardness of EPDM composites across various aging temperatures. Their findings indicated an increase in hardness by approximately 3.7% during the initial stages of aging at 125 °C, followed by further escalation during subsequent stages of aging. After aging at 180 °C for 7 days, the hardness rapidly increased by 37% [15]. These performance changes in EPDM vulcanizates were attributed to the extensive oxidation of the ethylene and propylene units within the EPDM macromolecular chains during thermo-oxidative aging [16].

However, the literature on the aging performance and service life prediction of spacer rubber composites is limited. This study primarily focused on spacer damping rubber composites and conducted aging tests under various conditions. Subsequently, an analysis was conducted to assess the changes in the performance of rubber damping composites upon aging. Furthermore, we have formulated a predictive model for estimating the service life of spacer rubber composites, which can guide the selection of suitable damping materials for power transmission applications.

## 2. Experimental Materials and Methods

### 2.1. Materials

The spacer damping rubber compound comprised commercial EPDM composites, which is commercially available as HL3310. In the following discussion, we will uniformly use EPDM for commercialized products. The formula for the EPDM composites is shown in Table 1. 

### 2.2. Methods

#### 2.2.1. Preparation of EPDM Specimens

The commercially available spacer damping rubber compound was mixed on a two-roll open mill, with the roll distance of the open mill adjusted to 2 mm for sheet production. The rubber compound was then placed into a preheated mold and hot-pressed on a vulcanizing machine to prepare test samples with different properties. The EPDM composites were vulcanized for the optimum curing time at 150 °C and 15 MPa.

#### 2.2.2. Characterization

The chemical structures of rubber composites were identified by Fourier transform infrared spectrometry (TENSOR 27, Bruker, Karlsruhe, Germany) in the ATR mode. The wavenumber ranged from 3500 to 600 cm^−1^ at a scanning resolution of 4 cm^−1^ and each spectrum was scanned 32 times. The thermal stability test was carried out on a thermogravimetric analysis instrument (TGA2, METTLER TOLEDO, Zurich, Switzerland). The test atmosphere was N_2_ atmosphere, with a heating rate of 10 K/min and a testing temperature range from 40 to 800 °C. The surface morphology of rubber composites was observed using a scanning electron microscope (TESCAN CLARA, TESCAN, Brno, Czech Republic). The test samples were coated with a gold thin film before observation to avoid charging during SEM testing. 

The crosslinking density of rubber composites before and after the thermal aging was measured by the equilibrium swelling method [17]. The rubber samples of about 0.5 g were immersed into cyclohexane for 72 h, during which the solvent was replaced with fresh cyclohexane every 24 h. The crosslinking density was calculated according to the Flory–Rehner equation [18]:

(1) $$V_{e}=-\frac{\ln(1-V_{r})+V_{r}+\chi V_{r}^{2}}{V_{s}(V_{r}^{1/3}-\frac{1}{2}V_{r})}$$

where *V_e_* is the crosslinking density (mol/cm^3^), *V*_r_ is the volume fraction of rubber, *V*_s_ is the molar volume of the solvent, *χ* is the interaction between EPDM and cyclohexane with a value of 0.35 [19]. The value of *V*_r_ is obtained according to the following equation:

(2) $$V_{r}=\frac{(m_{2}-m_{0}\varphi )\rho_{s}}{(m_{2}-m_{0}\varphi )\rho_{s}+(m_{1}-m_{2})\rho_{r}}$$

where *V*_r_ *m*_0_ is the mass of the rubber sample before swelling, *m*_1_ is the mass of the rubber after swelling, *m*_2_ is the mass of the rubber after drying, *φ* is the mass fraction of fillers, and *ρ*_r_ and *ρ*_s_ are the solvent densities of EPDM and cyclohexane, respectively.

The curing characteristics of rubber compounds were tested using a vulcanizer (MR-C3, Beijing Ruida Yuchen Instrument Co., Ltd., Beijing, China) with a compound weight of approximately 5 g at different temperatures to obtain the optimum curing condition. The Shore hardness was tested with a Shore A type hardness meter (HTS-800A, Shanghai Yizong Precision Instrument Co., Ltd., Shanghai, China) according to the ISO 48-4:2018 standard [20]. The thickness of specimen was 6 mm. The testing was measured at different positions on the specimen, and the hardness value for each specimen was calculated based on five positions. The tensile properties were measured on an electronic tensile machine (CMT4104, New Sansi Material Testing Co., Ltd., Shenzhen, China) according to ISO 37:2017 [21]. The aged EPDM vulcanized sheets were cut into dumbbell-shaped specimens with dimensions of 115 mm × 6 mm × 2 mm and the gage length of the samples was 25 mm. The tensile test was carried out at a speed of 500 mm/min and the average value of five samples was taken. 

The dynamic mechanical performance was determined using a dynamic thermomechanical analyzer (Q800, TA Instruments, New Castle, DE, USA) in the tensile mode at a frequency of 25 Hz over the temperature range from −80 °C to 80 °C with a heating rate of 3 °C min^−1^. 

The compression set (CS) of rubber composites was tested using a compression permanent deformation tester (MZ4020, Jiangsu Mingzhu Testing Machinery Co., Ltd., Yangzhou, China) at different temperatures (60 °C, 70 °C, 80 °C, 90 °C, and 100 °C) under 25% applied strain according to the ISO 815-1:2019 standard [22]. The aged samples were cylinders with a diameter of 29.0 mm and a height of 12.5 mm. The aging time of samples at each temperature was set to 24 h, 48 h, 96 h, 168 h, 336 h, and 672 h. The CS value was calculated according to the following equation:

(3) $$CS=\frac{h_{0}-h_{1}}{h_{0}-h_{s}}\times 100\%$$

where *h*_0_ and *h*_1_ are the height of specimens before and after aging, respectively, and *h*_s_ is the height of the limiter.

The thermal oxidative aging test was conducted on an aging testing machine (GT-7017-ELU, Gotech Testing Machines Inc., Qingdao, China) according to ISO 188:2023 standard [23]. The specimens were subjected to accelerated aging tests at the five temperature points 60 °C, 70 °C, 80 °C, 90 °C, and 100 °C.

## 3. Results and Discussion

### 3.1. Chemical Structure of EPDM Composites

**Figure 1:**
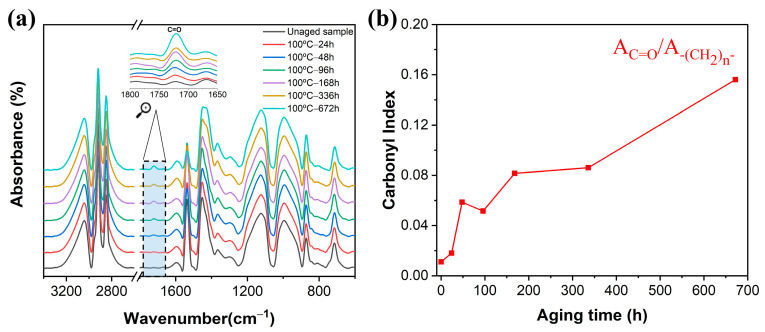
FTIR spectra (**a**) and carbonyl index (**b**) of EPDM composites under thermal oxidative aging at different times.

The aging of rubber composites refers to the occurrence of free radical chain reactions within rubber molecules under exposure to elevated temperature or oxygen [5]. This process results in the breaking of molecular chains or excessive crosslinking, ultimately leading to alterations in the properties of the rubber composites [5,6]. The chemical structures of spacer composites before and after aging were investigated by FTIR, as shown in Figure 1a. The peaks at 2918 cm^−1^ and 2847 cm^−1^ were attributed to the symmetric/asymmetric stretching vibrations of methylene (-CH_2_-) of EPDM composites, respectively [24]. In comparison, a distinct absorption peak emerged at 1720 cm^−1^ of aged EPDM composites ascribed to the formation of C=O group, with peak intensity serving as a measure of the oxidative degradation degree of the EPDM [16]. With increasing aging time, the peak at 1720 cm^−1^ corresponded to strengthened C=O groups [25,26]. The peaks around 1450 cm^−1^ and 1376 cm^−1^ were assigned to -CH_2_ and C-H vibrations of -CH_3_ from the propylene unit [27]. The peak at 872 cm^−1^ corresponded to the deformation vibrations of the typical unsaturated C=C-H in the diene portion, while the peak at 989 cm^−1^ represented the shear deformation vibration of the C-H bond in the third monomer, containing a double bond C=C-H [13]. The peak at 713 cm^−1^ was assigned to the in-plane rocking vibration of -(CH_2_)_n_- (n ≥ 5) owing to the presence of ethylene sequences in the EPDM backbone [28]. In order to assess the oxidative degradation degree of EPDM composites, the carbonyl index was determined by calculating the peak intensity of C=O at 1720 cm^−1^ with the peak at 713 cm^−1^ of -(CH_2_)_n_- groups. The carbonyl index of aged EPDM composites as a function of aging time is shown in Figure 1b. The results indicated that the carbonyl index significantly increased with the prolongation of aging time, suggesting gradual oxidation and degradation of EPDM vulcanizates [29].

### 3.2. Thermogravimetry Analysis of EPDM Composites

**Figure 2:**
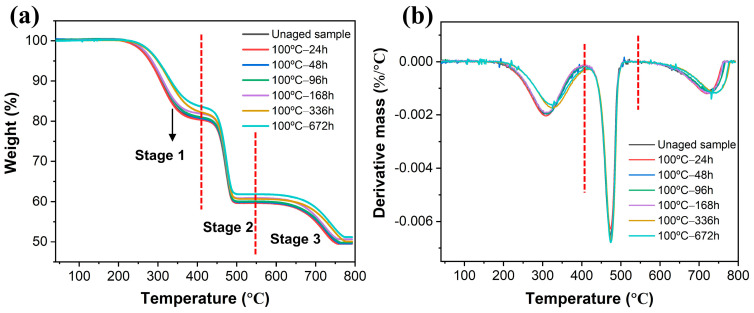
(**a**) TG curves and (**b**) DTG curves of rubber composites aged in various conditions.

Thermogravimetric analysis (TGA) serves as a valuable tool for evaluating the thermal stability of rubber composites [30]. Figure 2 shows the thermogravimetry (TG) and derivative thermogravimetry (DTG) curves of EPDM composites aged in various conditions. It was observed that degradation occurred in three distinct stages of EPDM composites. The first stage of weight loss, observed within the range of 200–400 °C, was attributed to the degradation of non-rubber components in EPDM composites, such as plasticizers, accelerators, and anti-aging agents [13]. The weight loss in this stage decreased with the prolongation of aging time, primarily due to the volatilization of small molecules during the high-temperature aging stage. The second stage, occurring within the temperature range of 400–550 °C, was attributed to the degradation of the EPDM matrix [31,32]. The DTG curve depicted in Figure 2b demonstrates that the aging of EPDM had negligible influence on the degradation temperature. The third stage, observed within the temperature range of 650–750 °C, could be attributed to the thermal decomposition of CaCO_3_ [33]. The residual substances included carbon black and the decomposition product of CaCO_3_ and ZnO mineral additives. The above results indicated that the thermal oxidative aging of EPDM mainly affected the weight loss in the first stage, with the volatilization and migration of small molecular substances further influencing the properties of rubber materials, as analyzed in the subsequent performance section.

### 3.3. Microstructure of EPDM Composites

**Figure 3:**
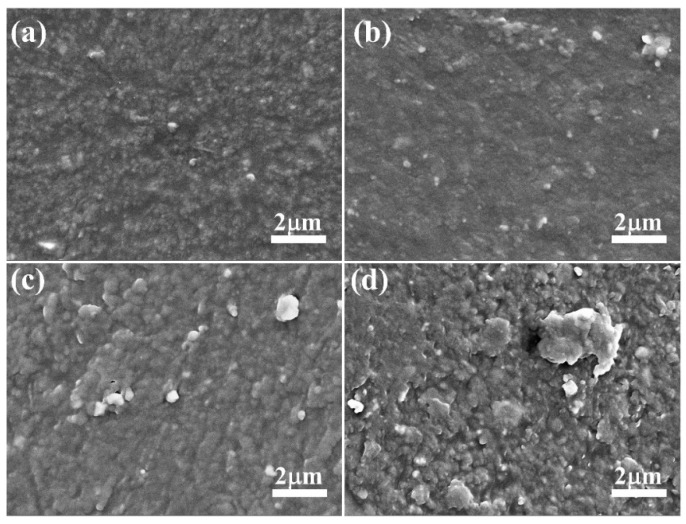
SEM images of surface morphology of EPDM composites: (**a**) unaged sample, (**b**) aged at 60 °C for 672 h, (**c**) aged at 80 °C for 672 h, (**d**) aged at 100 °C for 672 h.

The surface morphology of EPDM composites caused by thermal aging was observed by scanning electron microscopy (SEM). As shown in Figure 3, the surface of the unaged sample was relatively homogeneous and smooth, while that of the aged samples exhibited increased roughness. Moreover, as the aging temperature increased, numerous aggregates were observed on the surface of EPDM composites. At an aging temperature of 100 °C, larger aggregates of several microns in size appeared on the surface of the EPDM composites. These observations suggest that small molecular substances, such as plasticizers and antioxidants, migrate onto the surface during thermal oxidative aging, leading to the formation of aggregates on the surface [34,35,36,37]. Therefore, the migration of small molecular substances within the rubber matrix contributed to the deterioration of the mechanical properties of the rubber composites.

### 3.4. Mechanical Properties of EPDM Composites

**Figure 4:**
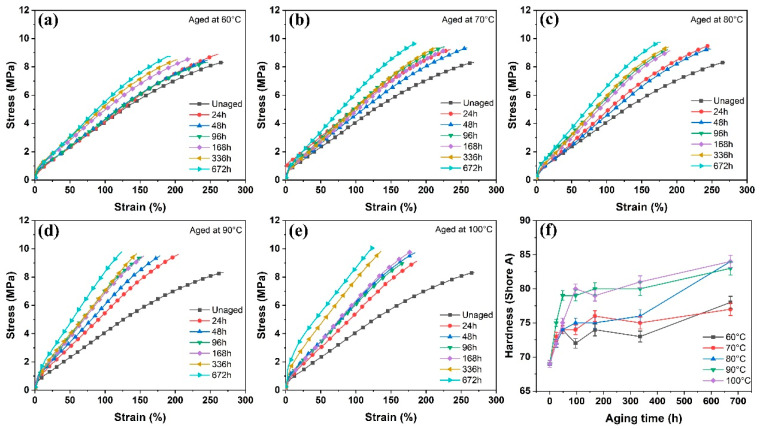
Mechanical properties and hardness of EPDM composites under different aging conditions: (**a**) Stress-strain curves aged at 60 °C, (**b**) Stress-strain curves aged at 70 °C, (**c**) Stress-strain curves aged at 80 °C, (**d**) Stress-strain curves aged at 90 °C, (**e**) Stress-strain curves aged at 100°C, (**f**) Hardness aged in various conditions.

The mechanical properties of rubber composites play a vital role in guaranteeing long-term stability of the product. Figure 4a–e present the stress–strain curves of EPDM composites subjected to aging at temperatures ranging from 60 °C to 100 °C. Compared with the unaged sample, the tensile strength slightly increased and exhibited a fluctuating trend over aging time with increasing aging temperature. Meanwhile, the elongation at the break of rubber composites gradually decreased with the increase in aging time and temperature. This decrease became more pronounced in elongation at break when the aging temperature surpassed 90 °C. Upon aging for 672 h, the elongation at break was reduced to 46.6% of its initial value. The tensile deformation of EPDM composites was affected by the increase in crosslinking density after aging, which inhibited the sliding of rubber molecular chains under external tension [29,38]. Consequently, the rubber molecular chains failed to fully absorb and dissipate external tensile energy during stretching, leading to a significant decrease in the elongation at break.

The curves demonstrated a gradual increase in modulus at 100% elongation of the EPDM composites with increasing aging time and temperature. Aging led to the formation of new crosslinks in rubber composites, restricting the movement of the rubber molecular chains and consequently resulting in a substantial increase in the modulus at 100% elongation [39]. After being subjected to 100 °C for 672 h, the modulus at 100% elongation of the aged sample showed a 112% increase compared to the unaged sample.

Figure 4f presents the change in Shore A hardness of EPDM composites before and after aging. With prolonged aging time, the hardness of the rubber sample generally increased at a fixed aging temperature. When the aging time remained constant, an increase in aging temperature resulted in an overall upward trend in the hardness of the test sample, although some test points may exhibit fluctuations. When the aging temperature exceeds 90 °C, the hardness of the rubber sample significantly increased after aging, suggesting that elevated temperatures accelerated the aging of the EPDM composites, resulting in rapid performance degradation.

### 3.5. Crosslinking Density of EPDM Composites

**Figure 5:**
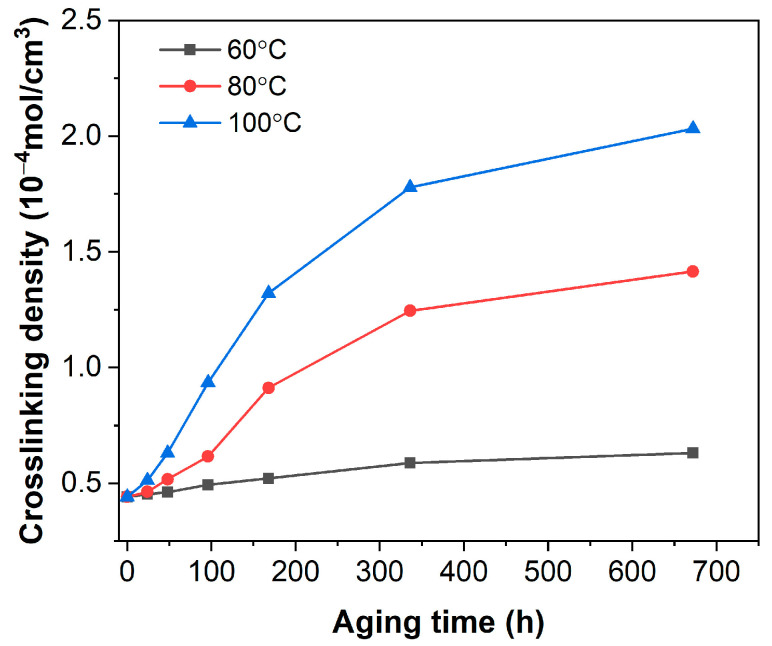
Crosslinking density of EPDM composites before and after aging.

Figure 5 illustrates the crosslinking density of spacer rubber composites as a function of aging time at different temperatures. Compared with the unaged sample, the crosslinking density of EPDM composites gradually increased after aging. The crosslinking density of EPDM composites increased gradually with prolonged aging time at the same aging temperature. Similarly, at the same aging time, an increase in aging temperature led to a rapid rise in crosslinking density. Thus, both aging time and temperature can impact the crosslinking density of rubber materials. After aging, the crosslinking density of rubber materials increased due to chain breakage at high temperatures, followed by the formation of new crosslinking bonds through active free radicals [16,29].

### 3.6. Compression Set of EPDM Composites

**Figure 6:**
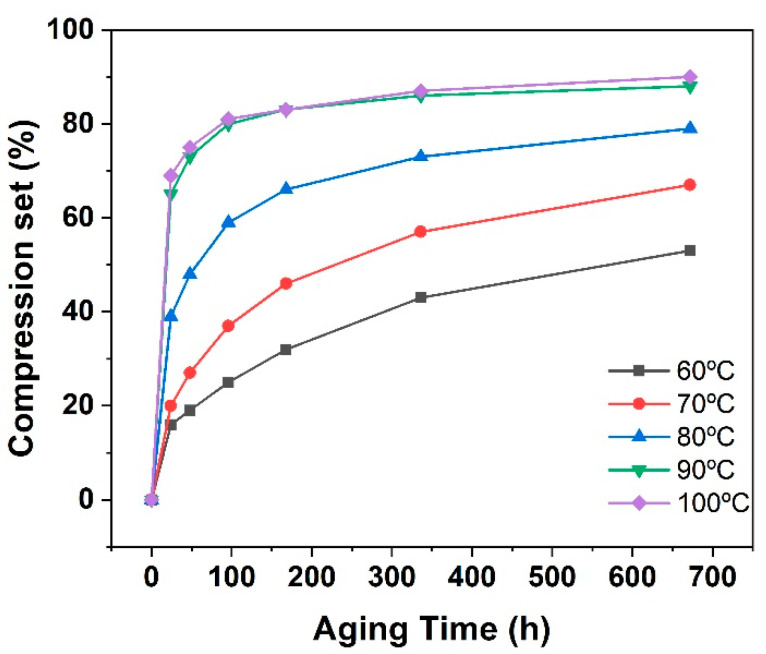
Compression set of spacer rubber composites before and after aging.

The compression set is an important indicator of the recovery performance of rubber composites after being compressed [40]. Figure 6 shows the compression set of rubber composites before and after aging. It is observed that under the same aging temperature, the compression set of the EPDM composites increased with prolonged aging time. Moreover, as the aging temperature increased, the EPDM composites exhibited a larger compression set. Given that the spacer rubber composites must withstand pressure for a long time during service life, the external force accelerated the aging failure of the EPDM composites. For instance, the rubber composites showed a 53% permanent deformation after exposure at 60 °C for 672 h.

### 3.7. Dynamic Damping of Rubber Composites

**Figure 7:**
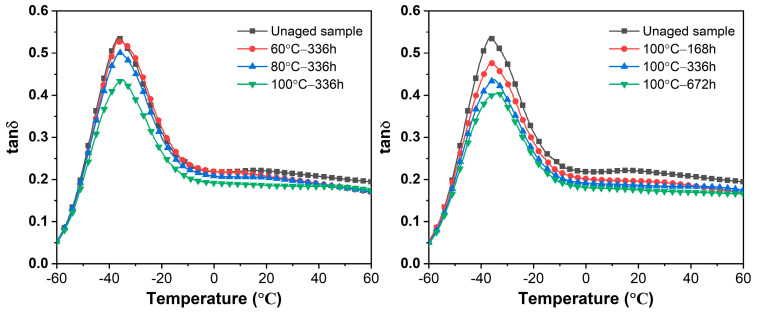
Dynamic damping of EPDM composites before and after aging.

Damping performance can be used to describe the damping and vibration reduction effect of rubber composites [41,42], commonly characterized by the loss factor (tan δ) [43]. Figure 7 presents the tan δ-temperature curves of the EPDM composites at different aging temperatures and times. The aged EPDM composites exhibited lower peak values of tan δ compared to the unaged sample, owing to the restricted mobility of the rubber molecular chains [44]. When the aging temperature remained constant, the tan δ gradually decreased with the aging time. After aging at 100 °C for 672 h, the tan δ at 20 °C reduced by 20.6% compared to the unaged sample. These experimental results showed that the aging of rubber composites affected the mutual mobility of rubber molecular chains, leading to reduced toughness and damping performance [45]. Consequently, the deterioration of the EPDM composites within the spacer diminished energy absorption during wire swinging and hindered the wire dancing control.

### 3.8. Service Life Prediction

Estimates of the service life of rubber composites for spacer bars can aid in optimizing rubber formulation, thereby providing better guidance and suggestions for their use [46]. The Arrhenius equation is a commonly used model for studying the relationship between chemical reaction rate and temperature. The relationship between aging reaction rate and temperature is normally described by the Arrhenius equation [47,48].

(4) $$K(T)=Ae^{\left(-E/RT\right)}$$

where *K(T)* is the reaction rate, *A* is a constant, *E* is the activation energy, *R* is the gas constant, and *T* is the absolute temperature. 

For rubber composites, the correlation between the performance variations of rubber materials at a specific temperature and time can be described as follows:

(5) f(P)=K(T)t

where *f(P)* is the performance after the aging reaction and *t* is the aging time. The reaction rate at any temperature is determined by observing the change in the selected property overtime at that specific temperature.

By combining and simplifying Formulas (4) and (5), the final formula is described as follows:

(6) $$\ln t=\ln\frac{f(P)}{A}+\frac{E}{RT}$$

**Figure 8:**
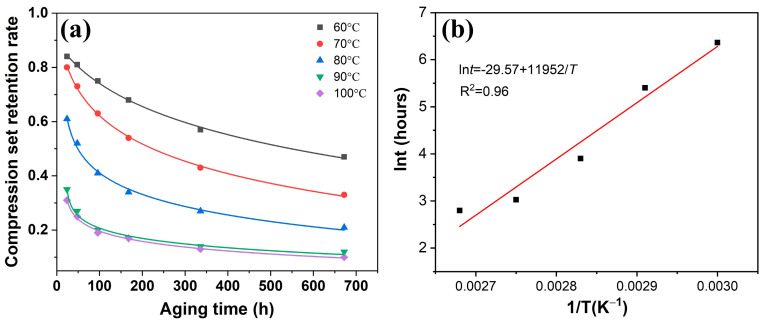
Compression set and aging life prediction for EPDM composites: (**a**) Compression set retention rate aged in various conditions, (**b**) lnt versus 1/T curves.

Further simplifying Formula (6), the relation lifetime and temperature relationship can be represented via Equation (7):

(7) $$\ln t=B+\frac{E}{RT}$$

where *B* is a constant. As shown in Figure 8, the value of *B* is −29.57 and the value of *E* is 99.4 kJ/mol.

As depicted in Figure 8a, the compression set is plotted against time at five temperatures. The compression set of EPDM composites exhibited a rapid increase during the early stage of aging, followed by a slowdown in the rate of compression set in the later stage, ultimately stabilizing. According to previous research, when the compression set retention reached 50%, the rubber composites were considered to have failed [49]. Figure 8b shows a linear relationship between ln*t* and *T*^−1^ in the life prediction equation, with a coefficient of determination *R*^2^ of 0.96, indicating a high degree of fitting. Using the derived equation, the service life of the EPDM composites for the spacer was determined at different temperatures. With increasing usage temperature, the predicted service life of EPDM composites gradually decreased. For instance, at a usage temperature of 20 °C, the service life of commercially available EPDM composites was estimated to be 8.4 years. Therefore, the establishment of a lifetime prediction model can provide guidance for the use of spacer rubber composites.

## 4. Conclusions

In this study, the aging performance of EPDM composites for spacer bars was studied. The results revealed that the rubber molecular structure of the spacer bar rubber composites gradually changed during thermal oxidative aging. With increasing aging time, the peak at 1720 cm^−1^ corresponded to C=O groups strengthened, suggesting a more severe oxidation and degradation of EPDM vulcanizates. Moreover, the Shore A hardness and modulus at 100% elongation of rubber composites significantly increased with an increase in aging temperature or the extension of aging time, accompanied by a notable decrease in elongation at break. This phenomenon occurred primarily due to the degradation of rubber molecular chains, leading to the formation of new crosslinked structures during aging. Furthermore, the damping performance of EPDM composites significantly decreased with aging. After aging at 100 °C for 672 h, the tan δ at 20 °C had decreased by 20.6% compared to the unaged sample. The irreversible movement of rubber molecular chains under external force post-fracture resulted in a large permanent deformation. Additionally, to estimate the lifespan of EPDM composites used in spacer bars, a life prediction model was developed based on compression set measurements. This model estimated that at an ambient temperature of 20 °C, the service life of EPDM composites can reach 8.4 years. Overall, the establishment of a comprehensive life prediction model for specific rubber composites used in spacer bars offers valuable technical support for spacer product services.

## Figures and Tables

**Table 1:** The formula of EPDM composites.

Chemicals	Contents (Per Hundred Parts of Rubbers, Phr)
EPDM	100
Zinc oxide	5
Stearic acid	3
Antioxidant 4010NA	3
Antioxidant RD	2
Carbon black (N550)	145
Calcium carbonate	115
Paraffin oil	80
Accelerator BZ	1
Accelerator TMTD 1.5	1.5
Accelerator M	1
Sulfur	1.5

## Data Availability

The data presented in this study are available on request from the corresponding author. The data are not publicly available due to privacy reasons.

## References

[1] Yang Z., Guo B., Zhang L. (2017). Challenge of rubber/graphene composites aiming at real applications. Rubber Chem. Technol..

[2] Lenko D., Schlögl S., Bichler S., Lemesch G., Ramsauer F., Ladstätter W., Kern W. (2015). Flexible epoxy-silicone rubber laminates for high voltage insulations with enhanced delamination resistance. Polym. Compos..

[3] Zhao Z., Liu B., Liu S., Zhang D. (2023). Design and Test of Quad-Bundle Spacer Damper Based on a New Rubber Structure. Shock Vib..

[4] Johnson B.L., Cameron F.K. (1933). Mechanism of rubber aging. Ind. Eng. Chem..

[5] Li G.-Y., Koenig J.L. (2005). A Review of Rubber Oxidation. Rubber Chem. Technol..

[6] Deuri A.S., Bhowmick A.K. (1987). Aging of EPDM rubber. J. Appl. Polym. Sci..

[7] Andrews R.D., Tobolsky A.V., Hanson E.E. (1946). The theory of permanent set at elevated temperatures in natural and synthetic rubber vulcanizates. J. Appl. Phys..

[8] Tobolsky A.V., Prettyman I.B., Dillon J.H. (1944). Stress relaxation of natural and synthetic rubber stocks. Rubber Chem. Technol..

[9] Dunn J.R., Scanlan J., Watson W.F. (1959). Stress relaxation during the thermal oxidation of vulcanized natural rubber. Trans. Faraday Soc..

[10] Bartenev G.M., Lyalina N.M. (1972). Stress relaxation mechanisms in rubbers reinforced with carbon blacks. Rubber Chem. Technol..

[11] Beatty J.R., Juve A.E. (1950). Stress relaxation of some rubber and synthetic rubber vulcanizates in compression. Rubber Chem. Technol..

[12] Ronan S. (2009). A Novel Approach to Predicting the Lifetime of Elastomers Undergoing Stress Relaxation. Ph.D. Thesis.

[13] Hu Q., Chen Q., Song P., Gong X., Chen J., Zhao Y. (2023). Performance of Thermal-Oxidative Aging on the Structure and Properties of Ethylene Propylene Diene Monomer (EPDM) Vulcanizates. Polymers.

[14] Delor-Jestin F., Lacoste J., Barrois-Oudin N., Cardinet C., Lemaire J. (2000). Photo-, thermal and natural ageing of ethylene–propylene–diene monomer (EPDM) rubber used in automotive applications. Influence of carbon black, crosslinking and stabilizing agents. Polym. Degrad. Stab..

[15] Kwak S.B., Choi N.S. (2011). Thermo-oxidative degradation of a carbon black compounded EPDM rubber hose. Int. J. Automot. Technol..

[16] Ning N., Ma Q., Zhang Y., Zhang L., Wu H., Tian M. (2014). Enhanced thermo-oxidative aging resistance of EPDM at high temperature by using synergistic antioxidants. Polym. Degrad. Stab..

[17] Tu J., Shi X., Jing Y., Zou H., Kadlcak J., Yong Z., Liu S., Liu G. (2021). Relationships of tensile strength with crosslink density for the high-carbon black-filled EPDM compounds with various softeners. Polym. Eng. Sci..

[18] Paul J., John R. (1943). Statistical mechanics of cross-linked polymer networks. I. Rubberlike elasticity. J. Chem. Phys..

[19] Baranwal K.C., Lindsay G.A. (1972). Diene Termonomer Type and EPDM Properties. Rubber Chem. Technol..

[20] (2018). Rubber, Vulcanized or Thermoplastic Determination of Hardness Part 4: Indentation Hardness by Durometer Method (Shore Hardness).

[21] (2017). Rubber, Vulcanized or Thermoplastic—Determination of Tensile Stress-Strain Properties.

[22] (2019). Rubber, vulcanized or thermoplastic—Determination of compression set—Part 1: At ambient or elevated temperatures.

[23] (2023). Rubber, Vulcanized or Thermoplastic Accelerated Ageing and Heat Resistance Tests.

[24] Alexander M., Thachil E.T. (2006). A comparative study of cardanol and aromatic oil as plasticizers for carbon-black-filled natural rubber. J. Appl. Polym. Sci..

[25] Zhao Q., Li X., Gao J. (2008). Surface degradation of ethylene–propylene–diene monomer (EPDM) containing 5-ethylidene-2-norbornene (ENB) as diene in artificial weathering environment. Polym. Degrad. Stab..

[26] Zhao Q., Li X., Gao J. (2007). Aging of ethylene–propylene–diene monomer (EPDM) in artificial weathering environment. Polym. Degrad. Stab..

[27] Li C., Wang Y., Yuan Z., Ye L. (2019). Construction of sacrificial bonds and hybrid networks in EPDM rubber towards mechanical performance enhancement. Appl. Surf. Sci..

[28] Gunasekaran S., Natarajan R., Kala A. (2007). FTIR spectra and mechanical strength analysis of some selected rubber derivatives. Spectrochim. Acta Part A.

[29] Li C., Ding Y., Yang Z., Yuan Z., Ye L. (2020). Compressive stress-thermo oxidative ageing behaviour and mechanism of EPDM rubber gaskets for sealing resilience assessment. Polym. Test..

[30] Gamlin C., Markovic M.G., Dutta N.K., Choudhury N.R., Matisons J.G. (2000). Structural Effects on the Decomposition Kinetics of EPDM Elastomers by High-Resolution TGA and Modulated TGA. J. Therm. Anal. Calorim..

[31] Tomer N.S., Delor-Jestin F., Singh R.P., Lacoste J. (2007). Cross-linking assessment after accelerated ageing of ethylene propylene diene monomer rubber. Polym. Degrad. Stab..

[32] Liu J., Wang B., Zhang L.W., Zhu L., Luo T.Y. (2015). Thermal aging behavior of ethylene propylene diene monomer (EPDM) rubber. Appl. Mech. Mater..

[33] Longkaew K., Gibaud A., Tessanan W., Daniel P., Phinyocheep P. (2023). Spherical CaCO_3_: Synthesis, Characterization, Surface Modification and Efficacy as a Reinforcing Filler in Natural Rubber Composites. Polymers.

[34] Liu J., Li X., Xu L., Zhang P. (2016). Investigation of aging behavior and mechanism of nitrile-butadiene rubber (NBR) in the accelerated thermal aging environment. Polym. Test..

[35] Sarkar P., Bhowmick A.K. (2018). Sustainable rubbers and rubber additives. Polymer.

[36] Hakkarainen M., Albertsson A.-C., Karlsson S. (2003). Migration and emission of plasticizer and its degradation products during thermal aging of nitrile rubber. Int. J. Polym. Anal..

[37] Chen S., Li T., Wan S., Huang X., Cai S., He X., Zhang R. (2019). Effect of Nitrogen-Doped Graphene Oxide on the Aging Behavior of Nitrile–Butadiene Rubber. Polymers.

[38] Zhang X., Li J., Chen Z., Pang C., He S., Lin J. (2022). Study on Thermal-Oxidative Aging Properties of Ethylene-Propylene-Diene Monomer Composites Filled with Silica and Carbon Nanotubes. Polymers.

[39] Abdel-Aziz M.M., Basfar A.A. (2000). Aging of ethylene-propylene diene rubber (EPDM) vulcanized by γ-radiation. Polym. Test..

[40] Lou W., Zhang W., Liu X., Dai W., Xu D. (2017). Degradation of hydrogenated nitrile rubber (HNBR) O-rings exposed to simulated servo system conditions. Polym. Degrad. Stab..

[41] Hu X., Zhang R., Wemyss A.M., Du A., Bao X., Geng X., Wan C. (2022). Damping and Electromechanical Behavior of Ionic-Modified Brominated Poly(isobutylene-co-isoprene) Rubber Containing Petroleum Resin C5. Ind. Eng. Chem. Res..

[42] Bala A., Gupta S. (2021). Thermal resistivity, sound absorption and vibration damping of concrete composite doped with waste tire Rubber: A review. Constr. Build. Mater..

[43] Wang X., Chen X., Song M., Wang Q., Zheng W., Song H., Fan Z., Myat Thu A. (2020). Effects of Hindered Phenol Organic Molecules on Enhancing Thermo-Oxidative Resistance and Damping Capacity for Nitrile Butadiene Rubber: Insights from Experiments and Molecular Simulation. Ind. Eng. Chem. Res..

[44] Evgin T., Mičušík M., Machata P., Peidayesh H., Preťo J., Omastová M. (2022). Morphological, Mechanical and Gas Penetration Properties of Elastomer Composites with Hybrid Fillers. Polymers.

[45] Xu Z., Jerrams S., Zheng L., Zhang L., Liu L., Wen S. (2021). Green Fabrication of High-Performance, Lignosulfonate-Functionalized, and Reduced-Graphene Oxide Styrene–Butadiene Rubber Composites. Ind. Eng. Chem. Res..

[46] Xiang K.-L., Xiang P.-Y., Wu Y.-P. (2014). Prediction of the fatigue life of natural rubber composites by artificial neural network approaches. Mater. Des..

[47] Woo C.S., Park H.S. (2011). Useful lifetime prediction of rubber component. Eng. Fail. Anal..

[48] Guo X., Yuan X., Liu G., Hou G., Zhang Z. (2023). Storage Life Prediction of Rubber Products Based on Step Stress Accelerated Aging and Intelligent Algorithm. Polymers.

[49] Vakulov N.V., Myshlyavtsev A.V., Malyutin V.I. (2015). Estimation of in-use Guaranteed Rubber Lifetime test methods. Procedia Eng..

